# Long-acting pre-exposure prophylaxis preferences among pregnant and postpartum women in Kenya: results from a discrete choice experiment

**DOI:** 10.1016/j.xagr.2025.100494

**Published:** 2025-04-08

**Authors:** Tessa Concepcion, John Kinuthia, Felix A. Otieno, Eunita Akim, Brian P. Flaherty, Laurén Gómez, Grace John-Stewart, Emmaculate M. Nzove, Nancy Ngumbau, Jerusha N. Mogaka, Ben Odhiambo, Anjuli D. Wagner, Salphine Watoyi, Jillian Pintye

**Affiliations:** 1 Department of Global Health, University of Washington, Seattle, (Concepcion, John-Stewart, Wagner and Pintye).; 2 Research and Programs Department, Kenyatta National Hospital, Nairobi, Kenya, (Kinuthia, Otieno, Akim, Nzove, Ngumbau, Odhiambo, Watoyi and Pintye); 3 Department of Psychology, University of Washington, Seattle, (Flaherty).; 4 Department of Epidemiology, University of Washington, Seattle, (Gómez and John-Stewart).; 5 Department of Medicine, University of Washington, Seattle, (John-Stewart).; 6 Department of Pediatrics, University of Washington, Seattle, (John-Stewart).; 7 School of Nursing, University of Washington, Seattle, (Mogaka).; 8 Department of Biobehavioral Nursing and Health Informatics, University of Washington, Seattle, (Pintye).

**Keywords:** HIV prevention, maternal health, adherence, latent class analysis, preference heterogeneity, Injectable PrEP, vaginal ring PrEP, oral PrEP, antenatal care

## Abstract

**Background:**

New long-acting HIV pre-exposure prophylaxis (LA-PrEP) methods may address adherence barriers during pregnant and postpartum periods, when HIV risk is elevated. Understanding their preferences for LA-PrEP is essential for person-centered HIV prevention in maternal and child health (MCH) systems, yet evidence on preferred attributes is limited.

**Objective:**

To estimate pregnant and postpartum women’s preferred PrEP attributes using a discrete choice experiment (DCE) at important timepoints in the perinatal period

**Study design:**

From February 2023 to July 2024, we conducted a DCE among 513 HIV-negative pregnant and postpartum women taking daily oral PrEP in Kisumu and Siaya, Kenya, enrolled between 24–32 weeks gestation and a high HIV risk score. Participants completed the DCE with 12 choice sets at their third antepartum (median gestational age: 37.0 weeks) and/or 6-month postpartum visits. Attributes included effectiveness, form and dosing, safety data, side effects, collection place, cost, and multipurpose prevention (postpartum only). We fit effects-coded choice data to a conditional logit model, latent class analysis (LCA) for preference heterogeneity, and univariate multinomial logistic regressions to predict class membership by individual characteristics.

**Results:**

A total of 513 women completed the DCE at least once (151 at third antepartum, 509 at 6-month postpartum). Every 2-month injections were strongly preferred, showing the highest positive preference weight (pregnant: 1.22, 95% CI: 1.12–1.33; postpartum: 1.24, 95% CI: 1.18–1.30). Four latent classes emerged: “Flexible PrEP Adopters” (37.2%), “Safe and Effective Injection Preference” (16.5%), “Strong Injection Preference” (37.7%), and “Oral PrEP Preference” (8.6%). Higher parity was associated with lower odds of membership in “Flexible PrEP Adopters” (OR=0.6, 95% CI: 0.4–0.8, *P=*.001), “Safe and Effective Injection Preference” (OR=0.6, 95% CI: 0.4–0.8, *P=*.003), and “Strong Injection Preference” (OR=0.7, 95% CI: 0.5–1, *P=*.027) compared to “Oral PrEP preference.”

**Conclusions:**

Strong preferences for every 2-month injectables emphasize the need to prioritize LA-PrEP in this population. ANC settings can support diverse PrEP preference profiles with tailored counseling to account for individual preferences, PrEP experience, and obstetric history.

**AJOG Global Reports at a Glance**

**Why was this study conducted?**

○ Women in East and Southern Africa face higher HIV risk during pregnancy and postpartum compared to nonpregnant and nonpostpartum periods.
○ Adherence to daily oral PrEP is challenging due to pregnancy side effects and demands of motherhood during postpartum.
○ Long-acting PrEP methods like dapivirine vaginal rings and CAB-LA could improve adherence.
○ Understanding the preferences of pregnant and postpartum women for long-acting PrEP methods can shape person-centered HIV prevention

**Key findings**

○ Every 2-month injectable PrEP was the most preferred, with the highest preference weight in both antepartum and postpartum.
○ Four preference profiles were identified: Oral PrEP Preference, Flexible PrEP Adopters, Safe and Effective Injection Preference, and Strong Injection Preference
○ Women with more pregnancies were less likely to prefer injectables.

**What does this add to what is known?**

○ First stated preference study among pregnant and postpartum women evaluating PrEP options.
○ Heterogeneous preferences indicate that a 1-size-fits-all approach to PrEP delivery may not be optimal.

## Introduction

In settings with high HIV burden, incidence of HIV among pregnant and postpartum women is nearly double that of the general population.^1^ The World Health Organization (WHO) recommends daily tenofovir disoproxil fumarate (TDF)-based pre-exposure prophylaxis (PrEP) for pregnant and postpartum women at substantial risk for HIV in high-burden settings.^2^ However, adherence to daily oral PrEP remains a challenge, with up to 50% of women discontinuing use during routine antenatal care.^3^^,^^4^ The unique challenges of pregnancy and postpartum – including side effects that overlap with pregnancy symptoms, changes in behaviors associated with HIV exposure, and the demands of motherhood during postpartum– may impact persistence with daily oral PrEP use.^5^, ^6^, ^7^, ^8^, ^9^, ^10^

Given these barriers, novel long-acting (LA)-PrEP methods, such as dapivirine vaginal ring, bi-monthly injectable cabotegravir (CAB-LA), and twice-yearly lenacapavir (LEN), may offer more attractive alternatives to daily oral PrEP and enhance PrEP persistence during periods of increased risk of acquiring HIV, like pregnancy and postpartum.^11^, ^12^, ^13^, ^14^ Yet, there is limited evidence around LA-PrEP preferences among pregnant women. While LA-PrEP formulations have demonstrated effectiveness, their distinct attributes and potential to improve adherence and acceptability during pregnancy and postpartum warrant further exploration.^15^ Understanding preferences of pregnant and postpartum women for these LA-PrEP methods is necessary to inform person-centered HIV preventive care in maternal and child health (MCH) systems. Discrete choice experiments (DCEs) are a robust approach used to assess product preferences,^16^ and are increasingly used to optimize delivery of novel HIV prevention methods.^17^, ^18^, ^19^, ^20^ DCEs enable policymakers to prioritize service components, accurately procure and forecast resource needs, and enhance user engagement.^20^

The aim of this study was to estimate pregnant and postpartum women’s preferred PrEP attributes using a DCE at important timepoints in the perinatal period with an overarching goal to inform health planning for delivering choice of PrEP products to pregnant/postpartum women.

## Methods

### Study setting and recruitment

This study was nested within a randomized controlled trial (RCT) of pregnant women at 5 public health facilities in Kisumu and Siaya counties, Kenya (NCT04472884). Women in the RCT initiated PrEP during routine ANC care, were followed through 9 months postpartum, and were randomized to either 2-way SMS PrEP adherence support (Mobile Solutions for Women’s and Children’s Health (mWACh) PrEP) or standard of care (SOC) PrEP adherence support. Facility selection and full eligibility criteria has been published.^21^ The facilities were in high HIV prevalence areas with established infrastructure and collaboration with the Kenyan Ministry of Health. Facilities enrolled 600 HIV-negative pregnant women (24–32 weeks gestation) receiving ANC. Eligibility included being HIV and tuberculosis negative, HIV risk score ≥6 (translating to HIV incidence 7.3/100 person-years),^22^ newly initiating PrEP during at their ANC enrollment visit, age ≥18, cell phone access, and planned to remain in the area and receive postpartum care at the clinic.

### Survey development

A DCE is a survey method that presents participants with hypothetical scenarios where they choose between alternatives with predefined features (attributes) and corresponding levels. We designed a DCE to identify PrEP attributes prioritized by pregnant and postpartum women. Attributes and levels were initially developed with input from study partners and staff in Kisumu, Kenya, which included nurses providing ANC-PrEP care, community members, and research managers. Clinic staff brainstormed factors influencing patients’ decisions to take PrEP or other medications, producing a list refined to 5 attributes for antepartum and 6 for postpartum. Attributes, levels, and images were further adapted through pilot testing with staff, with each attribute linked to 2–4 levels and corresponding figures (Table 1). In the context of this study, “side effects” are understood by participants as experiences related to a medication that are distinct from safety concerns for themselves or their fetus.

**Table 1:** DCE attributes and levels

Attribute	Levels
*Effectiveness*: How well the PrEP product works at preventing HIV. It is described as a percent.	1. 60% 2. 85% 3. 90% 4. 99%
*Form and dosing*: How the PrEP drug looks, size of the product, how much and how often it needs to be taken. It also describes what the packaging looks like.	1. Large oral pill taken once a day, comes in a pill bottle 2. Injection taken once every 2 months 3. Large oral pill, taken before and after HIV risk event (2 pills before sex, 1 pill 24 hours after sex, and 1 pill 48 hours after sex), comes in a blister pack. 4. Flexible vaginal ring inserted once every month
*Available safety data*: Data to support the safety of taking this PrEP product for pregnant and breastfeeding women. Safety and follow up data usually show that there is no harmful impact of the medication on the developing baby.	1. Little to no data on the effect of this medication on the baby 2. Data show this medication is safe for the baby
*Side effects*: This attribute describes side effects like dizziness, nausea, or weight gain that can be experienced when taking PrEP.	1. About 5kg weight loss 2. About 5kg weight gain 3. Nausea/vomiting 4. No side effects
*Collection place & cost*: Collection place describes where PrEP is available for individuals in need of it and cost is associated with some collection places. Some collection places offer PrEP for free.	1. Health facility (free) 2. Pharmacy (for an added cost) 3. ANC (free) 4. Home delivery (for an added cost)
*Multipurpose Prevention Technology (MPT)*: This attribute describes if the PrEP medication also prevents pregnancy, or if contraception is separate. This attribute is only used in the postpartum DCE.	1. This medication also prevents pregnancy 2. This medication does not prevent pregnancy

*Concepcion. Long-acting pre-exposure prophylaxis preferences among pregnant and postpartum women in Kenya: AJOG Glob Rep, 2025*.

### Survey design

We designed an unlabeled experiment using Lighthouse Studio Version 9.15.4 (Sawtooth Software), where alternatives were presented without predefined names or labels, allowing participants to base their choices solely on variations in attribute levels. Participants were presented with 12 choice tasks to minimize cognitive burden while maintaining design efficiency.^23^ In each task, participants were shown 3 hypothetical PrEP scenarios and asked to choose their preferred 1 or to choose “Would rather take no PrEP at all” (Supplement 1: Example choice task). Sample size was estimated using the following equation: NN>500c/ta^24^^,^^25^ where (t) is the number of choice questions asked, (a) is the number of alternatives, and (c) is the number of analysis cells (c). We used 3 alternatives (a) per choice set, 4 levels (maximum) per attribute (multiplied in order to investigate interactions 2×4=8), yielding 16 analysis cells (c), and 12 choice sets, suggesting a minimum sample size of 222.

### Data collection

The DCE was conducted at participants’ third antepartum study visit (∼32–40 weeks gestation) and 6 months postpartum, aligning with WHO exclusive breastfeeding guidelines.^26^ Data collection ran from February 2023 to July 2024. All participants initiated daily oral PrEP for the first time during pregnancy as part of the parent RCT, at least 2 months prior to completing the first DCE. The survey included an introduction to DCEs, with interviewers providing a definition of each PrEP attribute and its associated levels before participants answered 12 choice sets, each presenting 3 hypothetical PrEP options and a “no PrEP at all” choice. Data on demographics, PrEP use, reproductive health, and LA-PrEP interest was also collected as part of the clinical trial. Attributes were presented in a fixed order, with levels randomized using Sawtooth Software (Lighthouse Studio v9.15.4).

### Predictors of interest

Participants reported missed PrEP doses over the past 30 days, categorized as perfect adherence (0 missed doses) or non-adherence (≥1 missed dose). PrEP discontinuation was recorded if stopped at the current or any prior visit. Self-efficacy was measured using the 12-item PrEP Adherence Self-Efficacy Scale (PrEP-ASES).^27^ Depressive symptoms (CES-D score ≥10) ^28^^,^^29^ and anxiety (GAD-7: minimal ≤4, mild 5–9, moderate 10–14, severe ≥15),^30^ were assessed, both scales validated in Kenya.^30^^,^^31^ Perceived stress scale (PSS) was categorized as low, moderate, or high.^32^ Intimate partner violence (IPV) was identified using the HITS scale (score >10).^33^ Data were collected on previous pregnancy losses, high adverse childhood experiences (ACE score ≥6),^34^ education level (secondary or above), and infant outcomes, including live birth, prematurity (<37 weeks), and WHO weight-for-age z-score (WAZ), height-for-age z-score (HAZ), head circumference-for-age z-score (HCAZ), and weight-for-height z-score (WHZ).^35^

### Statistical analysis

Descriptive statistics summarized baseline and follow-up characteristics of participants who completed the DCE. Continuous variables were presented as medians and interquartile ranges (IQR), while categorical variables were summarized as frequencies and percentages. Participant preferences for PrEP attributes were estimated using a conditional logit model with effects coding. Results of this model are presented as preference weights (PW), which quantify the relative value participants assign to specific attribute levels, indicating how each level contributes to the overall perceived benefit of an option.^36^ Positive PW indicate higher preference for a level, while negative PW indicate lower preference. The model also estimated PW for selecting “no PrEP at all.” Overall importance at the attribute level was calculated as the range between the most- and least-preferred levels, divided by the total range of all attributes.^37^

We used latent class analysis (LCA) to assess preference heterogeneity.^38^ We fit models with 2 to ten classes and selected the optimal model based on adjusted Akaike Information Criterion (AIC), Bayesian Information Criterion (BIC), entropy, and scientific interpretability. To better understand the characteristics of individuals in each class, we ran univariate multinomial logistic regressions to predict class membership based on relevant characteristics from PrEP literature such as demographics, PrEP use, sexual behavior, obstetric history, and child outcomes. We adjusted models for age when the predictor variable was strongly correlated with age and could introduce confounding (e.g., number of pregnancies). Analyses were conducted using Sawtooth (Lighthouse Studio v9.15.4) and R (v4.3.0).

## Results

### Participant characteristics

A total of 513 women completed a DCE survey at least once, 151 in antepartum and 509 at postpartum visit. Among these, 147 women (28.7%) completed the DCE at both timepoints, 4 women (0.8%) only in antepartum, and 362 women (70.6%) only postpartum. The lower number of DCEs conducted in antepartum can be attributed to several factors, including missed visit or delivery before the third antenatal visit due to either early delivery or late gestational age at enrollment. The median gestational age at the antepartum DCE was 37.0 weeks (IQR: 36.2–38.4), and the median weeks postpartum at the postpartum DCE was 26.3 weeks (IQR: 26.0–27.0) (Table 2). Participants who completed the DCE at both antepartum and postpartum time points exhibited similar baseline characteristics. Both pregnant and postpartum participants had a median of 2.0 previous pregnancies (IQR: 1.0–3.0). Among postpartum participants, nearly all (99.0%, n=504) had a live birth. PrEP characteristics were assessed at the time of the DCE. At the third antenatal visit, 22.5% (n=34) of participants had discontinued PrEP, compared to 35.0% (n=178) at the 6-month postpartum visit. Among those still using PrEP, half missed 1 or more PrEP pills in the previous month, with 50.0% (n=58) during antepartum and 56.7% (n=187) during postpartum. Side effects from daily oral PrEP were reported by 20.3% (n=25) of antepartum participants and 15.4% (n=56) of postpartum participants.

**Table 2:** Demographic, PrEP taking, sociobehavioral, and obstetric characteristics of pregnant (N=151) and postpartum (N=509) women who completed a DCE on LA-PrEP preferences

	**Third antenatal follow-up**, N = 151^a^ Gestational weeks: 37.0 (36.2, 38.4)	**Six months postpartum**, N = 509^a^ Weeks postpartum: 26.3 (26.0, 27.0)
**Enrollment characteristics**		
Randomization arm		
Mobile PrEP adherence support	73 (48.3%)	257 (50.5%)
Standard of care	78 (51.7%)	252 (49.5%)
Age	24.0 (21.5, 29.0)	25.0 (22.0, 29.0)
Married	106 (70.2%)	359 (70.5%)
Secondary education or higher	97 (64.2%)	319 (62.7%)
Has regular employment	33 (21.9%)	106 (20.9%)
>2 people per room in household	42 (27.8%)	147 (28.9%)
High ACE score (Score ≥6)	40 (26.5%)	130 (25.5%)
**PrEP characteristics**		
Discontinued PrEP	34 (22.5%)	178 (35.0%)
Missed ≥1 PrEP pills last month^b^	58 (49.6%)	187 (56.5%)
Missing	1	1
Experienced side effects^b^	25 (21.4%)	52 (15.7%)
PrEP self-efficacy score^b^	8.7 (7.5, 10.0)	9.0 (7.7, 10.0)
**Sociobehavioral characteristics**		
Elevated depressive symptoms	2 (1.3%)	8 (1.6%)
Anxiety symptom score		
Minimal	151 (100.0%)	496 (97.4%)
Mild	0 (0.0%)	10 (2.0%)
Moderate	0 (0.0%)	3 (0.6%)
**Sexual behavior characteristics**		
Partner HIV status		
HIV negative	5 (3.3%)	28 (5.5%)
HIV positive	6 (4.0%)	15 (2.9%)
Unknown status	128 (84.8%)	436 (85.7%)
No partner	12 (7.9%)	30 (5.9%)
Number of lifetime sexual partners	3.0 (2.0, 4.0)	3.0 (2.0, 4.0)
Has ever exchanged sex for money	2 (1.3%)	18 (3.5%)
Has been diagnosed with or treated for an STI	2 (1.3%)	6 (1.2%)
**Obstetric history**		
Previous pregnancies	2.0 (1.0, 3.0)	2.0 (1.0, 3.0)
Previous pregnancy loss	20 (13.2%)	64 (12.6%)
Gestational age when starting PrEP	24.0 (24.0, 26.0)	26.0 (24.0, 29.0)
Currently on FP		240 (65.6%)
Missing		143^c^
Currently using injectable FP		58 (11.4%)
**Current pregnancy and child outcomes**		
Live birth		504 (99.0%)
Baby premature (<37 weeks)		17 (3.3%)
Low WAZ		22 (4.6%)
Missing		34
Low HAZ		33 (7.0%)
Missing		36
Low WHZ		30 (6.3%)
Missing		36
Currently breastfeeding		467 (92.7%)
Missing		5
Child ill since birth		77 (15.1%)
Child hospitalized since birth		4 (0.8%)

ACE, Adverse childhood experience; FP, family planning; STI, Sexually transmitted infection; WAZ, Weight-for-age z-score; HAZ, Heigh-for-age z-score; HCAZ, Head circumference-for-age z-score; WHZ, weight-for-height z-score.

a n (%); Median (IQR).

b among those who continued with PrEP.

c Data abstracted from PrEP card not available at this study visit. *Concepcion. Long-acting pre-exposure prophylaxis preferences among pregnant and postpartum women in Kenya: AJOG Glob Rep, 2025.*

### Participant preferences

**Figure 1:**
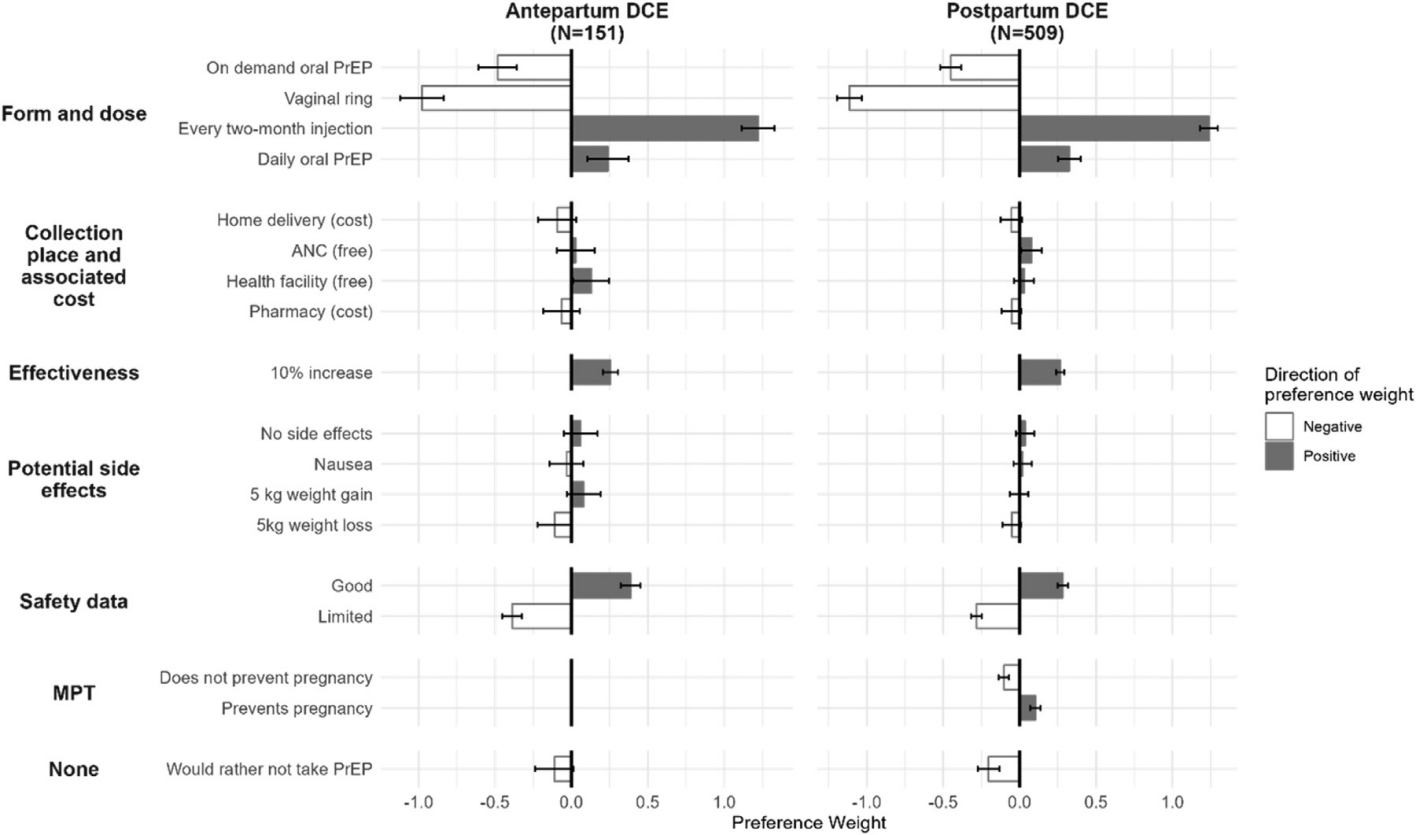
Positive (filled in) and negative (outlined) LA PrEP attribute utilities by perinatal stage.

Among both pregnant and postpartum participants, the every 2-month injection was strongly preferred, showing the highest positive PW (pregnant: 1.22, 95% CI: 1.12–1.33; postpartum: 1.24, 95% CI: 1.18–1.30; Figure 1). Good safety was the next highest preference, and PW was slightly higher among pregnant participants (0.39, 95% CI: 0.32–0.45) compared to postpartum participants (0.28, 95% CI: 0.25–0.31). A 10% increase in PrEP effectiveness also showed similar positive utilities (pregnant: 0.26, 95% CI: 0.21–0.30; postpartum: 0.27, 95% CI: 0.24–0.29). Daily oral PrEP had a PW of 0.24 (95% CI: 0.10–0.38) for pregnant participants and 0.32 (95% CI: 0.25–0.39) for postpartum participants. Overall, both groups disliked the vaginal ring (pregnant PW: -0.98, 95% CI: -1.12 to -0.83; postpartum PW: -1.11, 95% CI: -1.20 to -1.03) and event-driven oral PrEP (pregnant: -0.48, 95% CI: -0.61 to -0.36; postpartum: -0.45, 95% CI: -0.52 to -0.38). Additionally, both preferred some form of PrEP over none, as reflected by negative utilities for the option “Would rather not take PrEP” (pregnant: -0.11, 95% CI: -0.24 to 0.02; postpartum: -0.20, 95% CI: -0.27 to -0.14).

Concepcion. Long-acting pre-exposure prophylaxis preferences among pregnant and postpartum women in Kenya: AJOG Glob Rep, 2025.

For overall attribute importance, antepartum and postpartum groups both identified “form and dose” as the most important factor (antepartum: 50.2%, postpartum: 53.7%). “Effectiveness” ranked as the second most important attribute for both groups (antepartum: 22.7%, postpartum: 23.7%), followed by “safety data” (antepartum: 17.7%, postpartum: 12.9%). The next most important attribute for postpartum participants was “multipurpose prevention technologies (MPT)” with 4.7%. The least important attributes for both groups were “collection place and associated cost” (antepartum: 5.1%, postpartum: 3.0%) and “potential side effects” (antepartum: 4.3%, postpartum: 2.0%).

### Preference heterogeneity

**Figure 2:**
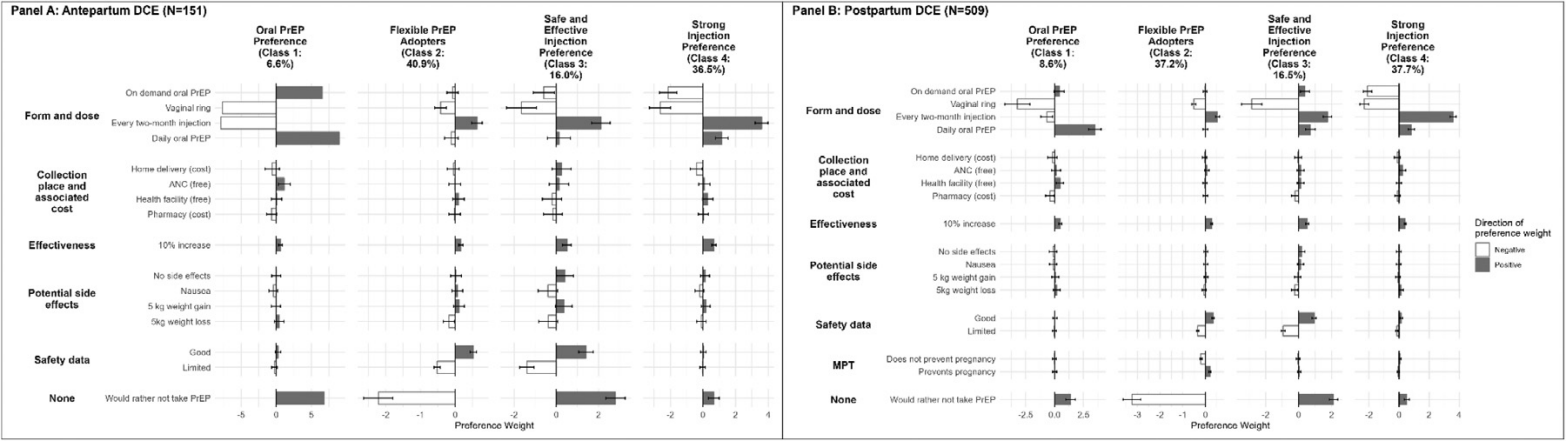
Positive (filled in) and negative (outlined) LA-PrEP attribute utilities by latent class among antepartum (Panel A) and postpartum (Panel B) participants.

A 4-class model was selected based on priori hypotheses and statistical tests (Supplement 2). The LCA results for antepartum and postpartum DCEs were comparable (Figure 2A and 2B, respectively). In the postpartum DCE, the first class, “Oral PrEP Preference,” comprised 8.6% of the sample. This group showed a strong preference for daily oral PrEP (PW: 3.49, 95% CI: 2.95–4.03) and choosing no PrEP at all (PW: 1.39, 95% CI: 0.99–1.79), with significant aversions to injectable PrEP (PW: -0.67, 95% CI: -0.91 to -0.43) and the vaginal ring (PW: -3.23, 95% CI: -3.78 to -2.68). The second class, “Flexible PrEP Adopters,” made up 37.2% of the sample. This group showed mixed preferences, with a slight preference for every 2-month injections (PW: 0.53, 95% CI: 0.39–0.67) but no strong aversion to other methods and a strongly negative PW for taking no PrEP at all (PW: -3.24, 95% CI: -3.43 to -3.06). The third class, “Safe and Effective Injection Preference,” which accounted for 16.5% of the sample, had a strong preference for every 2-month injectable PrEP (PW: 1.76, 95% CI: 1.63–1.89) or no PrEP at all (PW: 2.12, 95% CI: 1.99–2.25). This group placed high importance on a 10% increase in effectiveness (PW: 0.52, 95% CI: 0.43–0.61) and good safety data (PW: 0.94, 95% CI: 0.84–1.04). The largest class, “Strong Injection Preference,” consisted of 37.7% of the sample. This group had the highest preference for every 2-month injections (PW: 3.59, 95% CI: 3.48–3.71).

* To improve readability, confidence intervals are not shown in the antepartum DCE results for “On demand oral PrEP” (95% CI: -111.0 to 124.0), “daily oral PrEP” (95% CI: -109.0 to 127.0), and “would rather not take PrEP” (95% CI: -111.0 to 124.0) in Class 1. Confidence intervals for “vaginal ring” and “every 2-month injection” in Class 1 could not be estimated.Concepcion. Long-acting pre-exposure prophylaxis preferences among pregnant and postpartum women in Kenya: AJOG Glob Rep, 2025.

We used univariate multinomial logistic regression to determine which population characteristics were associated with higher odds of belonging to the “Flexible PrEP Adopters,” “Safe and Effective Injection Preference,” or “Strong Injection Preference” classes, compared to “Oral PrEP Preference” (Table 3). In the postpartum DCE, older participants had a lower odds of being in “Strong Injection Preference” (OR=0.9, 95% CI: 0.9-1.0, *P=*.021) compared to “Oral PrEP Preference.” Secondary education or higher was associated with higher odds of being in “Flexible PrEP Adopters” (OR=2.9, 95% CI: 1.5–5.6, *P=*.002), “Safe and Effective Injection Preference” (OR=3.1, 95% CI: 1.5–6.6, *P=*.003), and “Strong Injection Preference” (OR=2.0, 95% CI: 1.0–3.9, *P=*.036).

**Table 3:** Univariate^a^ associations of baseline, PrEP taking, and obstetric characteristics with latent class assignment among postpartum participants

	Frequency in sample^a^	Class 2 *(ref: Class 1)* *“Flexible PrEP adopters”*		Class 3 *(ref: Class 2)* *“Safe, effective injection”*		Class 4 *(ref: Class 2)* *“Strong injection preference”*	
		OR (95% CI)	p	OR (95% CI)	p	OR (95% CI)	p
**Enrollment characteristics**							
Received mobile adherence support	257 (50.5%)	0.6 (0.3–1.2)	.138	0.6 (0.3–1.3)	.227	0.7 (0.4–1.4)	.323
Age (years)	25.0 (22.0, 29.0)	1.0 (0.9–1.0)	.108	1.0 (0.9–1.1)	.951	0.9 (0.9–1.0)	.021
Married	359 (70.5%)	0.6 (0.2–1.4)	.210	0.4 (0.2–1.0)	.052	0.3 (0.1–0.7)	.009
Secondary education or higher	319 (62.7%)	2.9 (1.5–5.6)	.002	3.1 (1.5–6.6)	.003	2.0 (1.0–3.9)	.036
Has regular employment	106 (20.9%)	1.3 (0.6–2.6)	.482	0.8 (0.4–1.8)	.581	0.5 (0.2–1.0)	.061
> 2 people per room in household	147 (28.9%)	1.1 (0.5–2.7)	.824	2.7 (1.1–6.7)	.039	1.4 (0.6–3.4)	.426
High ACE score	130 (25.5%)	1.1 (0.6–2.3)	.710	1.0 (0.5–2.3)	.964	0.9 (0.4–1.8)	.683
**PrEP characteristics**							
Discontinued PrEP	178 (35.0%)	11.7 (3.5–39)	<.001	12.1 (3.5–42.2)	<.001	5 (1.5–16.9)	.009
Missed ≥1 pills in last month^b^	187 (56.5%)^b^	2.0 (1.0–4.2)	.067	1.9 (0.8–4.5)	.172	0.7 (0.3–1.4)	.295
Experienced side effects^b^	52 (15.7%)^b^	7.1 (2.3–22.3)	.001	3.8 (1.1–13)	.037	1 (0.4–2.1)	.909
PrEP self-efficacy score	9.0 (7.7, 10.0)^b^	0.7 (0.6–0.8)	<.001	0.8 (0.6–1.0)	.022	0.8 (0.6–1.0)	.030
**Sexual behavior characteristics**							
*Partner HIV negative (reference)*	28 (5.8%)	Ref		Ref		Ref	
Partner HIV positive	15 (3.1%)	0.6 (0.0–11.3)	.744	0.5 (0–10.3)	.653	0.3 (0–8.2)	.501
Partner status unknown	436 (91.0%)	0.3 (0.0–2.5)	.270	0.2 (0–1.7)	.144	0.7 (0.1–5.9)	.740
Number of lifetime sexual partners^a^	3.0 (2.0, 4.0)	1.1 (0.9–1.3)	.285	1.1 (1–1.3)	.115	1 (0.8–1.2)	.747
Has engaged in sex in exchange for money or other favors	18 (3.5%)	1.9 (0.2–16.0)	.536	1.7 (0.2–16.6)	.661	1.4 (0.2–12.0)	.752
Has been diagnosed with or treated for an STI	6.0 (1.2%)	0.5 (0.0–5.3)	.545	0.5 (0.0–8.9)	.669	0.5 (0.0–5.2)	.531
**Obstetric history**							
# of previous pregnancies^a^	2.0 (1.0, 3.0)	0.6 (0.4–0.8)	.001	0.6 (0.4–0.8)	.003	0.7 (0.5–1.0)	.027
Primigravida^a^	325 (63.9%)	2.0 (0.8–5.1)	.127	2.8 (1.0–7.9)	.046	2.1 (0.9–5.4)	.102
Previous pregnancy loss	64 (12.6%)	0.6 (0.3–1.5)	.272	0.7 (0.3–1.9)	.511	0.6 (0.3–1.5)	.296
# of previous live births^a^	2.0 (1.0, 2.0)	0.5 (0.4–0.8)	.003	0.6 (0.4–0.9)	.017	0.7 (0.5–1.0)	.057
# of children^a^	2.0 (1.0, 2.0)	0.5 (0.3–0.8)	.001	0.6 (0.4–0.9)	.017	0.6 (0.4–1.0)	.033
Currently using FP	240 (65.6%)	1.2 (0.6–2.5)	.592	1.7 (0.7–4.0)	.202	2 (1–3.9)	.058
Currently using injectable FP	58 (11.4%)	1.8 (0.5–6.1)	.383	1.1 (0.3–4.6)	.891	2.4 (0.7–8.2)	.171
Gestational age when starting PrEP	26.0 (24.0, 29.0)	0.9 (0.8–1.1)	.308	1 (0.8–1.1)	.583	0.9 (0.8–1.0)	.249
**Current pregnancy and child outcomes**							
Weeks postpartum	26.3 (26.0, 27.0)	1.1 (0.1–1.3)	.161	1.1 (0.9–1.4)	.204	1.2 (1–1.4)	.083
Baby born premature	19 (3.8%)	1.2 (0.1–10.7)	.860	2.9 (0.3–25.6)	.339	1.9 (0.2–15.7)	.546
Low WAZ	22 (4.6%)	1.3 (0.3–6.3)	.715	0.6 (0.1–4.5)	.623	1 (0.2–4.7)	.966
Low WHZ	30 (6.3%)	2.7 (0.3–21.6)	.352	1.8 (0.2–18.3)	.602	4 (0.5–31.4)	.181
Child ill since birth	77 (15.1%)	0.6 (0.2–1.3)	.174	0.6 (0.2–1.5)	.242	0.9 (0.4–2.0)	.772

1n (%); Median (IQR)

a Model included an adjustment for age when the predictor variable was strongly correlated with age and could introduce confounding

b Among those who continued on PrEPConcepcion. Long-acting pre-exposure prophylaxis preferences among pregnant and postpartum women in Kenya: AJOG Glob Rep, 2025.

Discontinuing daily oral PrEP was associated with higher odds of being in “Flexible PrEP Adopters” (OR=11.7, 95% CI: 3.5–39.0, *P<*.001), “Safe and Effective Injection Preference” (OR=12.1, 95% CI: 3.5–42.2, *P<*.001), and “Strong Injection Preference” (OR=5.0, 95% CI: 1.5–16.9, *P=*.009), relative to the “Oral PrEP Preference” class. Experiencing side effects from daily oral PrEP was associated with higher odds of being in the “Flexible PrEP Adopters” class (OR=7.1, 95% CI: 2.3–22.3, *P=*.001) and “Safe and Effective Injection Preference” class (OR=3.8, 95% CI: 1.1–13.0, *P=*.037), compared to the “Oral PrEP Preference” class.

Regressions for the number of previous pregnancies, being primigravida, number of previous live births and number of live children were adjusted for age. A higher number of previous pregnancies was associated with lower odds of being in “Flexible PrEP Adopters” (OR=0.6, 95% CI: 0.4–0.8, *P=*.001), “Safe and Effective Injection Preference” (OR=0.6, 95% CI: 0.4–0.8, *P=*.003), and “Strong Injection Preference” (OR=0.7, 95% CI: 0.5–1, *P=*.027), compared to the “Oral PrEP Preference” class. The number of previous live births, number of live children, and being primigravida had parallel results. None of the child outcomes, such as low WAZ, premature birth, or child illness since birth, were significantly associated with class membership.

In the antepartum DCE, receiving mobile adherence support was associated with a lower odds of being in the “Strong Injection Preference” class (OR: 0.1, 95% CI: 0.0–0.7, *P=*.019), compared to the “Oral PrEP Preference” class (Supplement 3).

## Discussion

### Principle findings

This DCE among pregnant and postpartum women in Kenya provides valuable insights into preferences for PrEP attributes. We found clear preferences for every 2-month injectable PrEP, which had the highest PW of any PrEP characteristic both in antepartum and postpartum. Other important attributes such as effectiveness and safety data underscore the importance of providing women with PrEP options that are both highly efficacious and supported by robust safety evidence. We identified 4 distinct preference profiles: Oral PrEP Preference, Flexible PrEP Adopters, Safe and Effective Injection Preference, and Strong Injection Preference, with the Strong Injection Preference group representing the largest proportion of the population. Flexible PrEP Adopters and Safe and Effective Injection Preference groups were open to multiple forms if safe and effective, while the largest class, Strong Injection Preference, strongly favored injectable PrEP but still had some positive views on daily oral PrEP. The smaller Oral PrEP Preference class strongly preferred daily oral PrEP or no PrEP, over injectables or the vaginal ring. The multinomial logistic regression analysis revealed several important predictors of class membership. Women who discontinued daily oral PrEP and had a higher number of previous pregnancies were more likely to belong to classes that favored injectables. The direction and magnitude of associations was similar among antepartum DCE participants, although small sample size limited our confidence intervals.

### Results in context

These findings align with previous studies indicating that PrEP options that are long-acting and less burdensome are preferred by women during antepartum and postpartum.^39^ A DCE conducted among postpartum women in South Africa and Zimbabwe similarly found that duration of protection was the most important feature of long-acting HIV prevention.^40^ Hetergenous preferences found in our latent class analysis reinforce the notion that a 1-size-fits-all approach to PrEP delivery may not be optimal. Membership in the smallest group, “Oral PrEP Preference,” may reflect a fear of injections, comfort with familiar options, or concerns about newer formulations.^41^^,^^42^ Most participants favored long-acting injectable PrEP. Bi-monthly injectables offer convenience and address challenges with daily adherence, particularly for pregnant and breastfeeding women managing pregnancy, newborn care, or postpartum recovery.^43^, ^44^, ^45^, ^46^, ^47^, ^48^ A South African study found postpartum women and those with prior pregnancies were 15% and 25% more likely to discontinue daily oral PrEP, respectively.^43^ Additionally, the discreet nature of injectables may appeal to women facing stigma or privacy concerns around HIV prevention.^49^^,^^50^

### Clinical implications

Current WHO guidelines recommend daily oral PrEP for women at substantial risk of HIV, including pregnant and lactating women.^2^ Although existing guidelines on long-acting HIV prevention methods, such as the dapivirine vaginal ring and CAB-LA, do not recommend use during pregnancy and lactation due to limited safety data,^51^^,^^52^ emerging evidence suggests a favorable safety profile,^53^^,^^54^ raising the possibility of future guideline updates to include pregnant and lactating women. Given that Kenyan national guidelines closely align with WHO recommendations, understanding preferences regarding long-acting PrEP is crucial for facilitating its clinical implementation in this context.^47^^,^^55^ This study highlights key implications for PrEP delivery in antenatal and postpartum care. The strong preference for every 2-month injectables underscores the need to prioritize LA-PrEP availability for pregnant and breastfeeding women, who require self-controlled, discrete HIV prevention options.^56^ The findings from this study indicate a potential high demand for long-acting injectable HIV PrEP, highlighting the need for clinics to strategically allocate resources and for providers to be equipped to counsel patients on emerging drug-drug interactions.^57^^,^^58^ We also found distinct preference classes and characteristics, such as oral PrEP experience and parity, that were associated with membership in specific preference classes. This data can be used to identify patients who may benefit most from new HIV prevention options. For example, we found that participants who discontinued oral PrEP were more likely to belong to a latent class that favored injections, highlighting that new long-acting injectable HIV PrEP could be particularly beneficial for certain subgroups. Additionally, healthcare providers can tailor counseling based on the predictors of latent class membership. For example, we found that patients who experienced oral PrEP side effects had higher odds of belonging to the “Safe and Effective Injection Preference” class compared to “Oral PrEP Preference” class, therefore providers may need to provide more comprehensive education about safety and side effects to this subgroup.

### Research implications

Decision support tools have been used to support family planning method choice^59^^,^^60^ and PrEP persistence among adolescent girls and young women in Kenya^61^ and could be a valuable implementation strategy to help women make informed choices and address the complexity of offering diverse PrEP options. In this DCE, we found an aversion to collection locations that were associated with a cost (home delivery or pharmacy) highlighting the importance of access to free PrEP.^62^ Postpartum women may face higher barriers to accessing PrEP, including transportation costs, logistical challenges, financial constraints, and lack of childcare, which make clinic visits burdensome and limit consistent access.^44^ Integrating LA-PrEP into routine maternal healthcare, particularly ANC clinics, has demonstrated acceptability and feasibility in Kenya and South Africa^4^^,^^63^^,^^64^ and can increase accessibility,^4^^,^^65^ reduce stigma,^63^ streamline care,^63^ improve adherence,^4^ and provide women with tailored support.^8^^,^^63^

### Strengths and limitations

There are several limitations to generalizability that should be considered. First, the DCE was conducted within a randomized trial which specifically enrolled women from ANC clinics, who took PrEP. Enrolling women from ANC clinics, potentially limits applicability to women who do not seek ANC services. The low preterm birth rate observed in this study (3.8%) may be attributed to participants receiving consistent antenatal care, direct access to nurses, and immediate follow-up for pregnancy concerns. Additionally, all participants had experience taking PrEP prior to the DCE. Thus, we are unable to capture decision-making processes of women who have not yet initiated PrEP. Participants were selected based on a high HIV risk score, which limits generalizability to populations with lower risk profiles. However, prioritizing PrEP for individuals at highest risk of HIV acquisition has the highest impact for reducing HIV incidence.^22^^,^^66^, ^67^, ^68^ Finally, this study was conducted prior to the clinical trial results of lenacapvir (LEN) for HIV prevention and, therefore, did not account for the form and dosing attribute of twice-yearly injections. Given the similarities in the administration methods between CAB-LA and LEN, along with the added benefit of a longer dosing schedule and higher efficacy for LEN, the finding in this study regarding the strong preference for an every 2-month injection could potentially be extrapolated to a 6-month injection schedule.

DCEs are valuable for understanding decision-making, though hypothetical scenarios may not fully reflect actual behavior. Despite this, there is evidence that stated preference can predict revealed behavior^69^ and this research advances person-centered care by uncovering the nuanced preferences and decision-making processes of diverse populations. Using preference data can enhance our ability to address disparities, align strategies with needs of historically marginalized communities, and facilitate the prioritization and evaluation of implementation determinants, ultimately contributing to more informed policy and program development.^70^

## Conclusion

We found that pregnant and postpartum women in Kenya strongly prefer an every 2-month injectable PrEP for HIV prevention, but there is a small subgroup that is characterized by a preference for oral PrEP. These findings contribute to the growing body of evidence supporting the need for multiple HIV PrEP options that align with women’s preferences during antepartum and postpartum. By incorporating women’s preferences, we can better meet their needs and ultimately improve HIV prevention outcomes.

## CRediT authorship contribution statement

**Tessa Concepcion:** Writing – review & editing, Writing – original draft, Visualization, Software, Project administration, Methodology, Funding acquisition, Formal analysis, Data curation, Conceptualization. **John Kinuthia:** Writing – review & editing, Supervision, Methodology, Funding acquisition, Formal analysis, Conceptualization. **Felix A. Otieno:** Writing – review & editing, Validation, Project administration, Methodology, Data curation, Conceptualization. **Eunita Akim:** Writing – review & editing, Validation, Project administration, Data curation, Conceptualization. **Brian P. Flaherty:** Writing – review & editing, Writing – original draft, Validation, Supervision, Software, Methodology, Formal analysis. **Laurén Gómez:** Writing – review & editing, Resources, Project administration, Investigation, Data curation. **Grace John-Stewart:** Writing – review & editing, Writing – original draft, Supervision, Methodology, Formal analysis, Conceptualization. **Emmaculate M. Nzove:** Writing – review & editing, Resources, Project administration, Investigation, Funding acquisition. **Nancy Ngumbau:** Writing – review & editing, Resources, Project administration, Investigation, Funding acquisition, Data curation. **Jerusha N. Mogaka:** Writing – review & editing, Project administration, Investigation, Data curation, Conceptualization. **Ben Odhiambo:** Writing – review & editing, Validation, Project administration, Investigation, Data curation, Conceptualization. **Anjuli D. Wagner:** Writing – review & editing, Validation, Methodology, Conceptualization. **Salphine Watoyi:** Writing – review & editing, Project administration, Data curation, Conceptualization. **Jillian Pintye:** Writing – review & editing, Writing – original draft, Validation, Supervision, Methodology, Investigation, Funding acquisition, Formal analysis, Conceptualization.

## Appendix Supplementary materials

Image, application 1
