# Supplementary material for: Long-acting pre-exposure prophylaxis preferences among pregnant and postpartum women in Kenya: results from a discrete choice experiment
Source: AJOG Glob Rep. 2025 Apr 8;5(2):100494. doi: 10.1016/j.xagr.2025.100494 (PMC12138433; doi:10.1016/j.xagr.2025.100494)
Supplement: Supplementary file 1 [file mmc1.docx]

# Supplemental Figure 1. Example DCE choice set for postpartum participants


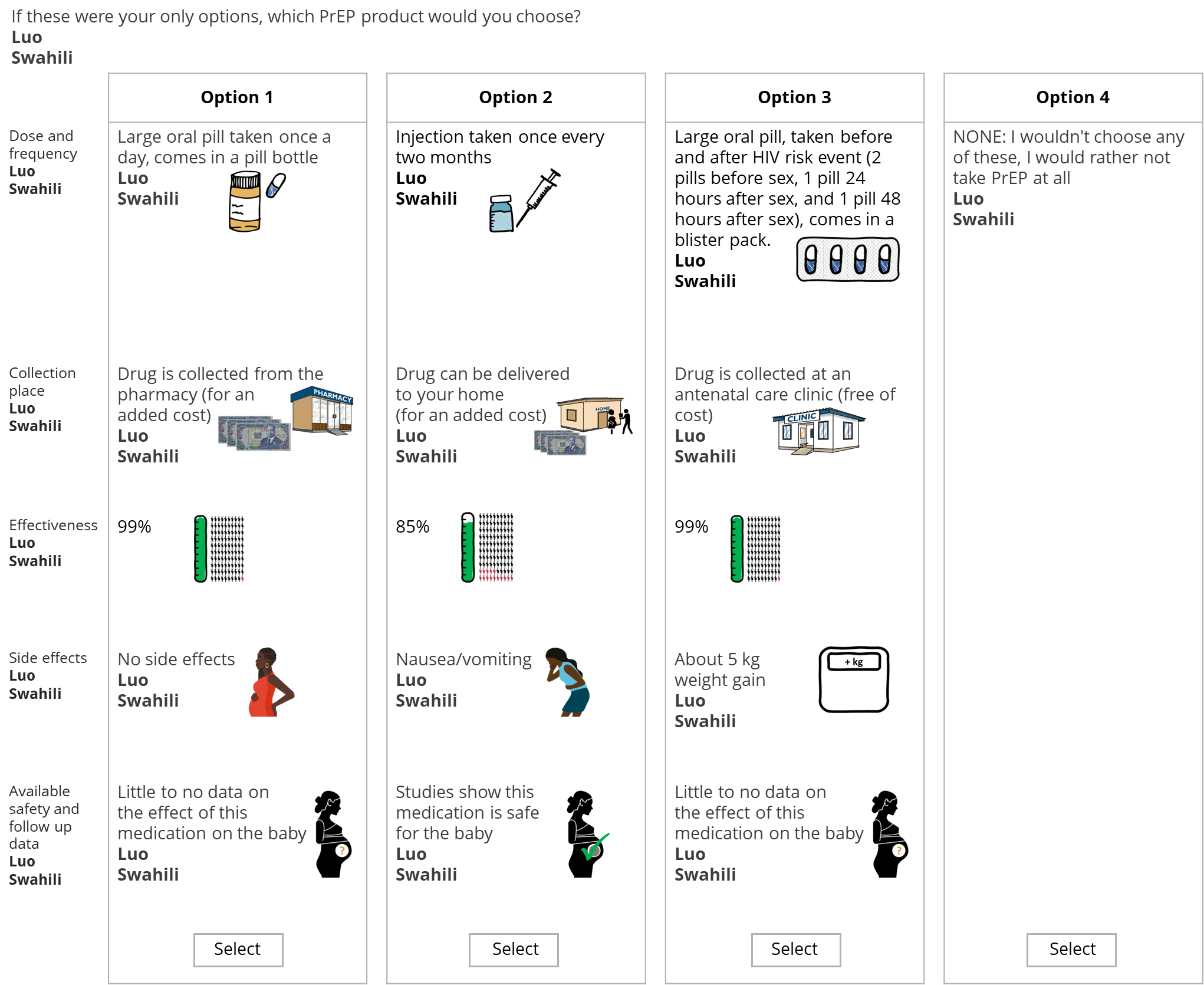


# Supplemental Table 2. Goodness of fit statistics for latent class models in antepartum (n=151) and postpartum (n=509) DCEs

| Classes | Log-likelihood | AIC | CAIC | BIC | ABIC | Chi-Square | Relative Chi-Square | Entropy |
| --- | --- | --- | --- | --- | --- | --- | --- | --- |
| Pregnancy | |  |  |  |  |  |  |  |
| 2 | -1793.75 | 3637.50 | 3800.02 | 3775.02 | 3695.59 | 1428.11 | 57.12 | 0.90 |
| 3 | -1663.40 | 3402.81 | 3649.83 | 3611.83 | 3491.10 | 1688.81 | 44.44 | 0.93 |
| 4 | -1577.89 | 3257.78 | 3589.31 | 3538.31 | 3376.29 | 1859.83 | 36.47 | 0.95 |
| 5 | -1523.63 | 3175.26 | 3591.30 | 3527.30 | 3323.97 | 1968.35 | 30.76 | 0.93 |
| 6 | -1480.98 | 3115.96 | 3616.50 | 3539.50 | 3294.87 | 2053.65 | 26.67 | 0.94 |
| 7 | -1439.15 | 3058.31 | 3643.35 | 3553.35 | 3267.43 | 2137.31 | 23.75 | 0.94 |
| 8 | -1411.27 | 3028.54 | 3698.10 | 3595.10 | 3267.87 | 2193.07 | 21.29 | 0.95 |
| 9 | -1377.22 | 2986.43 | 3740.49 | 3624.49 | 3255.97 | 2261.18 | 19.49 | 0.96 |
| 10 | -1369.78 | 2997.56 | 3836.13 | 3707.13 | 3297.31 | 2276.05 | 17.64 | 0.96 |
| Postpartum | |  |  |  |  |  |  |  |
| 2 | -6000.37 | 12062.74 | 12301.95 | 12270.95 | 12172.44 | 4920.37 | 158.72 | 0.90 |
| 3 | -5524.67 | 11143.34 | 11506.01 | 11459.01 | 11309.66 | 5871.77 | 124.93 | 0.92 |
| 4 | -5313.77 | 10753.54 | 11239.68 | 11176.68 | 10976.48 | 6293.57 | 99.90 | 0.94 |
| 5 | -5172.49 | 10502.97 | 11112.58 | 11033.58 | 10782.54 | 6576.14 | 83.24 | 0.94 |
| 6 | -5070.20 | 10330.40 | 11063.47 | 10968.47 | 10666.59 | 6780.71 | 71.38 | 0.93 |
| 7 | -4983.53 | 10189.06 | 11045.59 | 10934.59 | 10581.86 | 6954.05 | 62.65 | 0.92 |
| 8 | -4914.51 | 10083.02 | 11063.02 | 10936.02 | 10532.45 | 7092.08 | 55.84 | 0.91 |
| 9 | -4848.96 | 9983.93 | 11087.39 | 10944.39 | 10489.98 | 7223.18 | 50.51 | 0.91 |
| 10 | -4790.35 | 9898.69 | 11125.62 | 10966.62 | 10461.36 | 7340.42 | 46.17 | 0.92 |


# Supplemental Table 3. Full table of univariate* associations of baseline, PrEP taking, and obstetric characteristics with latent class assignment among antepartum participants (n=151)

|  | Frequency  in sample^1^ | Class 2 *(ref: Class 1)*  *“Flexible PrEP adopters”* | | Class 3 *(ref: Class 1)*  *“Safe, effective injection”* | | Class 4 *(ref: Class 1)*  *“Strong injection preference”* | |
| --- | --- | --- | --- | --- | --- | --- | --- |
|  |  | OR (95% CI) | p | OR (95% CI) | p | OR (95% CI) | p |
| **Enrollment characteristics** |  |  |  |  |  |  |  |
| Received mobile adherence support | 73 (48.3%) | 0.3 (0.1 - 1.5) | 0.151 | 0.2 (0 - 1.3) | 0.099 | 0.1 (0 - 0.7) | 0.019 |
| Age (years) | 24.0 (21.5, 29.0) | 1.0 (0.9 - 1.1) | 0.983 | 1.0 (0.9 - 1.2) | 0.848 | 1.0 (0.9 - 1.1) | 0.943 |
| Married | 106 (70.2%) | 0.3 (0 - 2.1) | 0.205 | 0.3 (0 - 2.4) | 0.233 | 0.2 (0 - 2) | 0.185 |
| Secondary education or higher | 97 (64.2%) | 1.4 (0.4 - 5.5) | 0.631 | 1.5 (0.3 - 7.1) | 0.593 | 1.0 (0.2 - 3.8) | 0.949 |
| Has regular employment | 33 (21.9%) | 0.5 (0.1 - 2.9) | 0.446 | 1.1 (0.2 - 7) | 0.911 | 2.1 (0.4 - 10.6) | 0.391 |
| > 2 people per room in household | 42 (27.8%) | 4.3 (0.5 - 36.2) | 0.181 | 2.5 (0.3 - 24.7) | 0.433 | 3.6 (0.4 - 30.8) | 0.242 |
| High ACE score | 40 (26.5%) | 4.6 (0.5 - 38.9) | 0.16 | 4.8 (0.5 - 45) | 0.169 | 2.0 (0.2 - 17.2) | 0.545 |
| **PrEP characteristics** |  |  |  |  |  |  |  |
| Discontinued PrEP | 34 (22.5%) | 2.9 (0.3 - 24.6) | 0.335 | 5.8 (0.6 - 53.8) | 0.123 | 1.7 (0.2 - 15.3) | 0.625 |
| Missed 1+ pills in last month** | 58 (49.6%)** | 5.4 (1.0 - 29.2) | 0.048 | 8.8 (1.2 - 61.7) | 0.029 | 2.2 (0.4 - 11.6) | 0.365 |
| Experienced side effects** | 25 (21.4%)** | 2.5 (0.5 - 12.1) | 0.256 | 8.0 (0.7 - 92.7) | 0.096 | 1.4 (0.3 - 6.4) | 0.676 |
| PrEP self-efficacy score | 8.7 (7.5, 10.0)** | 0.9 (0.6 - 1.2) | 0.352 | 0.9 (0.6 - 1.3) | 0.609 | 1.1 (0.8 - 1.6) | 0.442 |
| **Sexual behavior characteristics** |  |  |  |  |  |  |  |
| *Partner HIV negative (reference)* | 5 (3.6%) | Ref |  | Ref |  | Ref |  |
| Partner HIV positive | 6 (4.3%) | 3.1 (0 - >100) | 0.994 | >100 (0 - >100) | 0.96 | 3.1 (0 - >100) | 0.994 |
| Partner status unknown | 128 (92.1%) | 0 (0 - >100) | 0.914 | 12.1 (0 - >100) | 0.99 | 0 (0 - >100) | 0.916 |
| Number of lifetime sexual partners* | 3.0 (2.0, 4.0) | 1.1 (0.8-1.4) | 0.657 | 1.1 (0.8-1.4) | 0.488 | 1.1 (0.8-1.4) | 0.694 |
| Has engaged in sex in exchange for money or other favors | 2 (1.3%) | >100 (0 - >100) | 0.932 | 0 (0 - >100) | 0.993 | >100 (0 - >100) | 0.931 |
| Has been diagnosed with or treated for an STI | 2 (1.3%) | >100 (0 - >100) | 0.932 | 0 (0 - >100) | 0.993 | >100 (0 - >100) | 0.931 |
| **Obstetric history** |  |  |  |  |  |  |  |
| # of previous pregnancies* | 2.0 (1.0, 3.0) | 0.6 (0.3-1.2) | 0.170 | 0.6 (0.3-1.3) | 0.174 | 0.9 (0.5-1.7) | 0.709 |
| Primigravida* | 95 (62.9%) | 1.8 (0.3-10.2) | 0.519 | 2.9 (0.4-21.1) | 0.282 | 1.2 (0.2-7.2) | 0.806 |
| Previous pregnancy loss | 20 (13.2%) | 0.6 (0.1 - 3.3) | 0.551 | 0.4 (0 - 3.2) | 0.373 | 0.7 (0.1 - 3.7) | 0.644 |
| # of previous live births* | 2.0 (1.0, 3.0) | 0.7 (0.3-1.6) | 0.342 | 0.9 (0.3-2.4) | 0.861 | 1.1 (0.5-2.5) | 0.863 |
| # of children* | 2.0 (1.0, 3.0) | 0.6 (0.3-1.4) | 0.246 | 0.9 (0.3-2.3) | 0.813 | 1.1 (0.5-2.4) | 0.885 |
| Gestational age when starting PrEP | 24.0 (24.0, 26.0) | 0.9 (0.6 - 1.2) | 0.434 | 0.8 (0.6 - 1.3) | 0.424 | 0.9 (0.7 - 1.3) | 0.725 |
| *^1^*n (%); Median (IQR), *Model included an adjustment for age when the predictor variable was strongly correlated with age and could introduce confounding, **Among those who continued on PrEP | | | | | | | |
